# Distribution of bony erosions in feet and performance of two bone erosion scores: A dual-energy computed tomography study of 61 patients with gout

**DOI:** 10.1371/journal.pone.0259194

**Published:** 2021-11-02

**Authors:** Amandine Chabernaud Negrier, Lokmane Taihi, Eric Vicaut, Pascal Richette, Thomas Bardin, Frédéric Lioté, Hang-Korng Ea, Valérie Bousson

**Affiliations:** 1 Service de Radiologie, Hôpital Lariboisière, AP-HP.Nord-Université de Paris, Paris, France; 2 Unité de Recherche Clinique, Hôpitaux Lariboisière, Fernand Widal, Saint Louis, AP-HP.Nord-Université de Paris, Paris, France; 3 Service de Rhumatologie, INSERM UMR 1132, Hôpital Lariboisière, AP-HP.Nord-Université de Paris, Paris, France; University of Memphis, UNITED STATES

## Abstract

**Objectives:**

To assess the distribution of bone erosions and two erosion scores in the feet of patients with gout and analyze the association between erosion scores and monosodium urate (MSU) crystal deposition using dual-energy computed tomography (DECT).

**Materials and methods:**

We included all patients who underwent DECT of both feet between 2016 and 2019 in our radiology department, with positive detection of MSU deposits. Data on sex, age, treatment, serum urate, and DECT urate volumes were obtained. CT images were analyzed to score bone erosions in 31 sites per foot by using the semi-quantitative method based on the Rheumatoid Arthritis MRI Scoring (RAMRIS) system and the Dalbeth-simplified score. Reproducibility for the two scores was calculated with intraclass correlation coefficients (ICCs). Correlations between clinical features, erosion scores and urate crystal volume were analyzed by the Spearman correlation coefficient (r).

**Results:**

We studied 61 patients (mean age 62.0 years); 3,751 bones were scored. The first metatarsophalangeal joint and the midfoot were the most involved in terms of frequency and severity of bone erosions. The distribution of bone erosions was not asymmetrical. The intra- and inter-observer reproducibility was similar for the RAMRIS and Dalbeth-simplified scores (ICC 0.93 vs 0.94 and 0.96 vs 0.90). DECT urate volume was significantly correlated with each of the two erosion scores (r = 0.58–0.63, p < 0.001). There was a high correlation between the two scores (r = 0.96, p < 0.001).

**Conclusions:**

DECT demonstrates that foot erosions are not asymmetric in distribution and predominate at the first ray and midfoot. The two erosion scores are significantly correlated with DECT urate volume. An almost perfect correlation between the RAMRIS and Dalbeth-simplified scores is observed.

## Introduction

Gout is the most prevalent inflammatory arthritis worldwide [1]. It is a crystal deposition disease due to monosodium urate (MSU) crystals. Crystal deposits can induce acute inflammatory episodes and, at a late stage, tophi and erosive destructive arthropathies that are responsible for mechanical pain and disability. Early introduction of urate-lowering therapy (ULT) allows for dissolving MSU crystals and prevents the deleterious consequences of their chronic deposition [1].

Dual-energy computed tomography (DECT) has the remarkable ability to non-invasively detect MSU crystal deposits, with high sensitivity and specificity [2–10]. Classically, two energy spectra are produced, one at 80 kiloVolt peak (kVp) and the second at 135–140 kVp [11]. The combination of reconstructed images from raw data of each acquisition allows for detecting MSU crystal deposits and also provides an automatic quantification of the volume of these deposits. DECT also allows for obtaining bone images of high spatial resolution to precisely evaluate bone erosions, in an image quality similar to that provided by standard CT-scan acquisition. DECT is mainly used to detect tophi and for quantification, but its ability to additionally provide high resolution images has poorly been exploited. Indeed few studies [12–14] used DECT-based bone images reconstruction for analysis of gouty erosions.

Two CT bone erosion scores have been developed to quantify structural damage and capture the severity of the disease at the foot. The Rheumatoid Arthritis Magnetic Resonance Scoring (RAMRIS) system used for gout MRI scans [15] has been transposed to CT [16, 17]. A total of 31 bone sites per foot need to be scored, which involves a long reading time. Dalbeth et al. proposed a simplified model derived from the RAMRIS system [18, 19] focusing on only seven sites that are frequently affected and that are representative of gout damage in the foot [17].

Several studies have explored the relation between bone erosion and MSU crystal deposition. Histological and imaging studies have highlighted the close spatial relation between individual bone erosion and tophi [15, 20–24]. At the cellular level, osteoclasts are present at the interface between bone and tophi, and MSU crystals promote osteoclastogenesis [24] leading to bone erosion. One study found a correlation between the Dalbeth-simplified score and the foot urate volume (includes deposits adjacent and not adjacent to bone) [25]: the DECT urate volume explained 34% to 42% of the variance of the Dalbeth-simplified score (r = 0.58–0.65). However, to our knowledge, this result has not been reproduced, and the relation between the RAMRIS score and the DECT urate volume has not been investigated. In addition, an independent assessment of the Dalbeth-simplified score is lacking.

Therefore the objectives of our study were 1) to use DECT to assess the distribution of bone erosions and two erosion scores in feet of patients with gout, and 2) to analyze the relation between two CT bone erosion scores and urate volume.

## Materials and methods

### Study population

The institutional review board of our institution approved this retrospective study and granted waiver of informed consent (IRB 00006477- HUPNVS, University of Paris, AP-HP—Clinical trial registration number: NCT03965676). We selected from our Picture Archiving and Communication System all patients followed in the Rheumatology Department at Lariboisière Hospital (Paris, France) who underwent DECT for gout in the radiology department from November 2016 to March 2019 with the same DECT protocol and CT device. Inclusion criteria were 1) gout meeting American College of Rheumatology/European League Against Rheumatism diagnostic criteria [26], 2) undergoing DECT of both feet and ankles, and 3) non-artifactual detection of MSU crystals on DECT of feet and ankle. Sex, age, ULT and serum urate at the time of DECT were obtained from clinical records.

### DECT acquisition and image reconstruction

DECT was performed with a single-source 80-detector row scanner operating in double helical sequential acquisition mode with 135 kV/150 mA and 80 kV/600 mA (Aquilion Prime, Canon Medical Systems), without intravenous contrast material. Patients were positioned feet-first in a supine position. The scan was acquired in a craniocaudal direction, starting 5 cm from the ankle joint to the distal big toe. Both ankles and feet were scanned axially in one acquisition at 80 x 0.5 mm, field of view 320 mm, and pitch 0.638.

**Fig 1:**
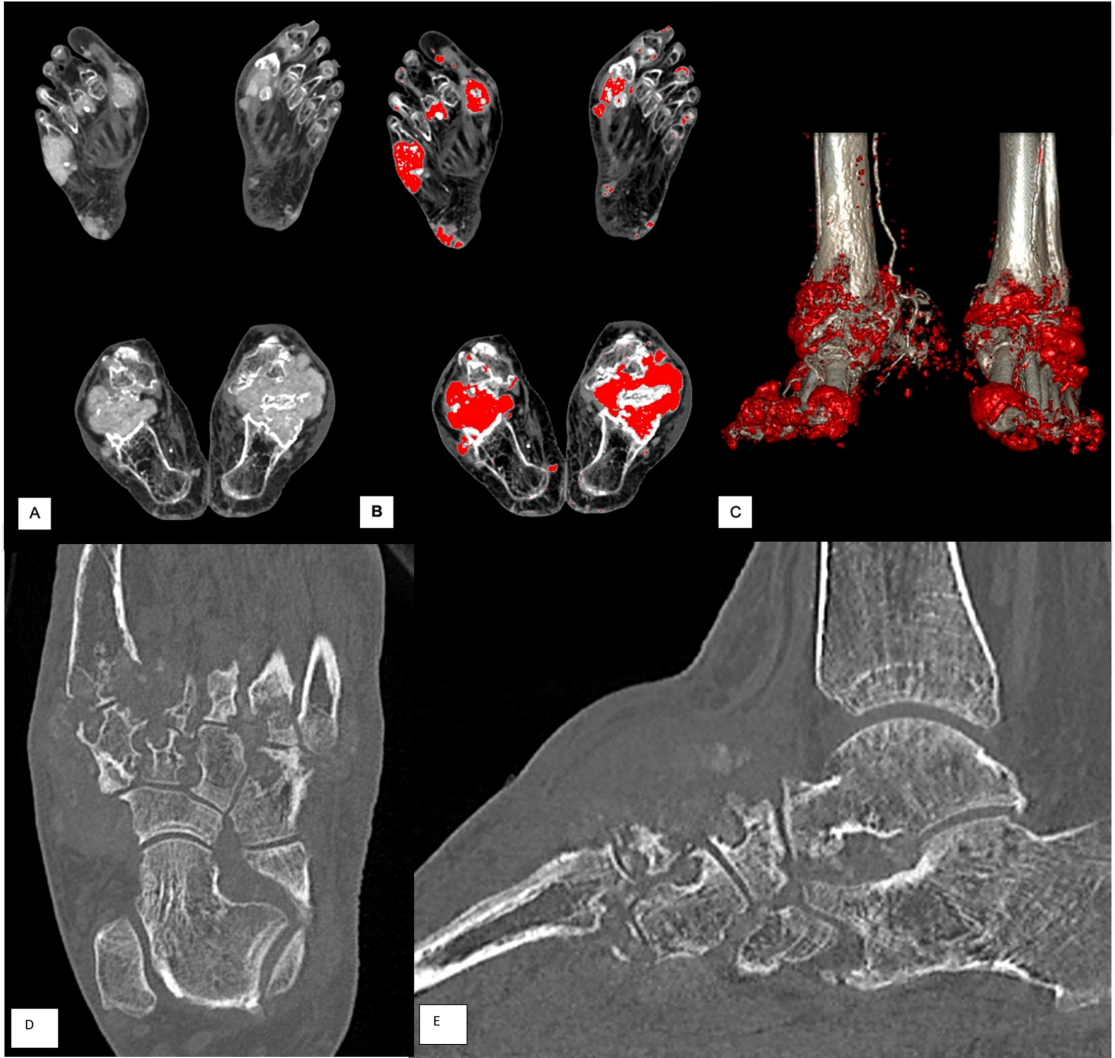
Dual-energy CT (DECT) examination of tophaceous foot. A: Soft filter reconstructed images. B: Color-coded composition CT images obtained with postprocessing techniques by using data from DECT show urate deposits in red areas within the tophi. C: Surface-rendered 3D CT images obtained with further postprocessing showing the anatomic relation between the MSU crystals–containing tophi (red areas) and bone structures (white areas). D and E: axial and sagittal bone filter reconstructed images to score bone erosions.

The images were reconstructed with iterative reconstruction on a bone algorithm and a soft-tissue algorithm, 512-pixel matrix, to a 0.50-mm slice with 0.3-mm increment. Acquisition and reconstruction parameters were optimized to obtain excellent spatial resolution for precise evaluation of bone erosions. A detailed description is in S1 Table. Fig 1 shows an example of DECT examination of a tophaceous foot.

### Image analysis

#### Bone erosion distribution and scores

One reader (ACN, a musculoskeletal radiology resident), with blinding to patient characteristics and DECT urate volume, assessed bone erosions by using CT reformatted images in the axial, sagittal and coronal planes and 3-D volume-rendering images. Assessment was performed after consensual training with two senior musculoskeletal radiologists (LT, a radiology fellow, VB, 20 years of experience, and ACN, interactively analyzed CT multiplanar reformations and scored bone erosions in ten tophaceous foot on a PACS workstation (Carestream)). Erosions were defined as focal areas of loss of cortex with sharply defined margins, seen in two planes, with a cortical break seen in at least one plane. Bone erosions were scored according to the RAMRIS-derived score [18, 19] and recorded separately to assess the distribution of bone erosions. The Dalbeth-simplified score [17] was also calculated.

*RAMRIS-derived score*. Each site was scored separately on a scale of 0 to 10 based on the proportion of eroded bone compared with the “assessed bone volume”, judged on all available images, with 0, no erosion; and 1, 1–10%; 2, 11–20%; and 10, 91–100% bone eroded [18, 19]. For long bones and large tarsal bones, the “assessed bone volume” was from the articular surface (or its best-estimated position if absent) to a depth of 1 cm. Bone erosions were assessed for 31 sites in each foot, corresponding to distal and proximal portions of the first to fifth proximal phalanges; first to fifth metatarsal (MT) heads; first to fifth MT bases; lateral, middle and medial cuneiforms; navicular, cuboid, anterior process of calcaneus; proximal calcaneus; distal talus; anterior talus; proximal talus; and distal tibia. Hence, all bones in the foot and ankle were evaluated except for the intermediate and distal phalanges and distal fibula.

Scores were also obtained per joint and per region for each foot for assessing erosion distribution. These regions were the forefoot (distal and proximal portions of the first to fifth proximal phalanges, first to fifth MT heads), the midfoot (first to fifth MT bases; lateral, middle and medial cuneiform; navicular; cuboid), and the hindfoot (anterior process of calcaneus and proximal calcaneus; distal, anterior and proximal talus; distal tibia).

*Dalbeth-simplified score*. The Dalbeth-simplified score was the sum of erosion scores from seven sites in each foot: first MT head, second MT base, third MT base, fourth MT base, cuboid, middle cuneiform, and distal tibia. The score was evaluated from 0 to 10 as for the RAMRIS-derived score.

*Intra- and interobserver agreement of the erosion score measurements*. The first reader (ACN) performed a second reading of bone erosions of 20 randomly selected DECT exams at 4 weeks after the initial reading, with blinding to the first scores. A second reader (LT) analyzed the same 20 randomly selected DECT exams with blinding to the first reader scores.

**Urate volume** was automatically determined by the dual energy software with the default settings of the CT-scan manufacturer (same settings for all analyses: material A is MSU; material B is bone; a constant partition line between MSU and bone; a constant threshold to exclude the tissue overlap regions; filter fonction to limit noise).

### Statistical analysis

Clinical features of study patients, DECT urate volume, and the two erosion scores are described with mean (standard deviation [SD]), median (Q1–Q3, interquartile range [IQR], range). Bone erosion distribution, erosion scores per bone, per region, per foot, per patient, and reading time are described with mean (SD), median (range) and prevalence defined as the proportion of non-null erosion score.

The symmetry (right/left) of the distribution of the erosion was evaluated by erosion score with a mixed ANOVA model, with bones, foot (left/right) and patients as random factors and by erosion prevalence according to the Cochran-Mantel-Haenszel test. Comparisons of clinical features and DECT variables by sex and by ULT or no treatment were evaluated by Fisher exact test, Student *t* test, or Wilcoxon-Mann-Whitney test according to variable distribution.

Correlations between DECT variables (erosion scores, urate volume) and age and serum urate were assessed by Spearman correlation coefficient.

Intra- and interobserver agreement for erosion scores was estimated by the intraclass correlation coefficient (ICC), with ICC 0–0.20 representing slight agreement, 0.21–0.40 fair agreement, 0.41–0.60 moderate agreement, 0.61–0.80 substantial agreement, and 0.81–1.00 near-perfect agreement [27].

All tests were two-sided, with p < 0.05 considered statistically significant. All analyses were performed with SAS, v9.2 (SAS, Cary, NC).

## Results

### Study population

**Fig 2:**
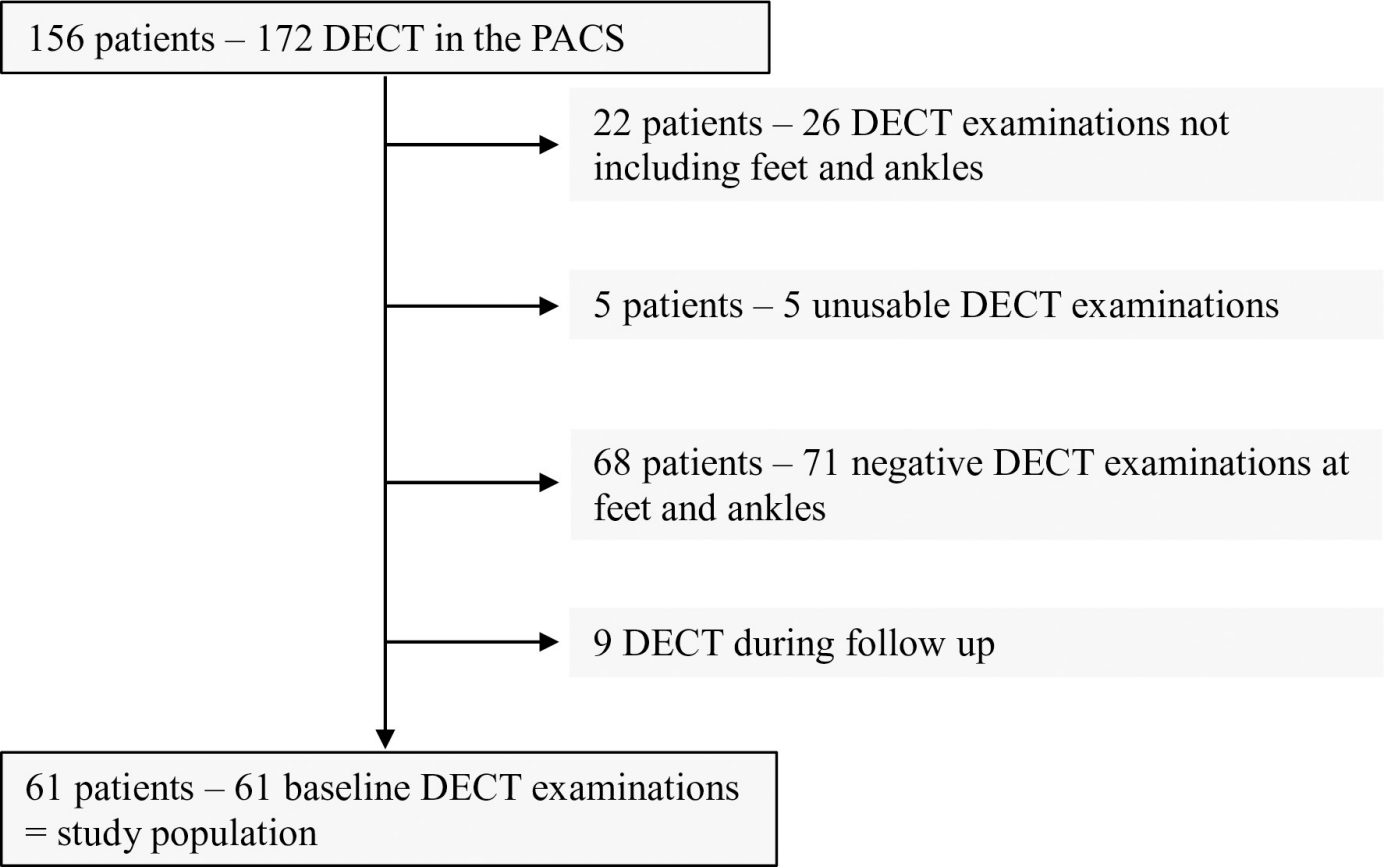
Flowchart of the inclusion of patients with gout. Footnotes: DECT: dual-energy computed tomography; PACS: Picture archiving and communication system.

During November 2016 to March 2019, 156 consecutive eligible patients underwent 172 DECT examinations; 61 patients with gout met the inclusion criteria and constituted our study population (Fig 2).

The study population is described in Table 1. The mean age was 62.0 ± 14.4 years; 49 were men. Mean serum urate was 464 ± 157 μmol/L. Overall, 23 patients were receiving ULT at the time of DECT (n = 9 allopurinol, n = 14 febuxostat), and their serum urate was significantly lower than those without ULT (p < 0.001). Only 13 of the ULT-treated patients had reached the target serum urate <6.0 mg/dL at the time of DECT. Mean DECT-measured urate volume was 9.25 ± 24.41 cc. DECT urate volume did not significantly differ by sex, age, or ULT status; it was weakly correlated with serum urate (r = 0.28, p = 0.04).

**Table 1:** Characteristics of the study population.

	Total
	N = 61
**Age, years**	
N (missing)	61 (0)
Mean±SD	62.0 ± 14.4
Median (Q1–Q3)	65.0 (56.0–72.0)
Range	17.0–88.0
**Serum urate, μmol/L**	
N (missing)	58 (3)
Mean±SD	464.5±157.8
Median (Q1–Q3)	488 (342–587)
Range	16–785
**Urate volume, ml**	
N (missing)	58 (3)
Mean±SD	9.25±24.41
Median (Q1–Q3)	1.39 (0.63–5.03)
Range	0.13–155.05

N: number; SD: standard deviation; Q: quartiles

### Erosion distribution and scores

Overall, 121 feet were examined (only one foot in one patient). Bone erosions for 3,751 sites were scored.

#### Distribution

Table 2 provides descriptive statistics concerning the RAMRIS erosion score for 31 sites per foot and for three regions: forefoot, midfoot, and hindfoot regions.

**Table 2:** Rheumatoid Arthritis MRI Scoring (RAMRIS) erosion scores and prevalence of gout erosions. 2A. RAMRIS erosion scores for each site ranked in decreasing order based on mean value, and prevalence of gout erosion of each site. 2B. RAMRIS erosion scores, and prevalence of erosion of each region and joint.

A: RAMRIS erosion scores for each bone ranked in decreasing order based on their mean value, and prevalence of gout erosion of each bone				
	**Bones**	**Mean score**	**Median (range)**	**Prevalence**
**1**	First MT head	1.24	1 (0–10)	0.71
**2**	Proximal portion of the first PP	0.94	1 (0–9)	0.57
**3**	Fourth MT base	0.91	0 (0–10)	0.47
**4**	Second MT base	0.80	1 (0–10)	0.59
**5**	Lateral cuneiform	0.80	1 (0–10)	0.64
**6**	Navicular	0.79	1 (0–8)	0.64
**7**	Distal portion of the first PP	0.73	0 (0–10)	0.36
**8**	Middle cuneiform	0.72	1 (0–5)	0.59
**9**	Medial cuneiform	0.69	1 (0–3)	0.64
**10**	Third MT base	0.68	0 (0–8)	0.50
**11**	Cuboid	0.67	1 (0–5)	0.56
**12**	Distal talus	0.59	0 (0–8)	0.45
**13**	Proximal calcaneus	0.55	0 (0–10)	0.43
**14**	Fifth MT base	0.53	0 (0–10)	0.35
**15**	Proximal talus	0.53	0 (0–3)	0.47
**16**	First MT base	0.51	0 (0–7)	0.41
**17**	Anterior process of calcaneus	0.47	0 (0–3)	0.42
**18**	Anterior talus	0.45	0 (0–8)	0.31
**19**	Fifth MT head	0.44	0 (0–10)	0.21
**20**	Distal tibia	0.42	0 (0–3)	0.39
**21**	Second MT head	0.42	0 (0–10)	0.17
**22**	Third MT head	0.28	0 (0–10)	0.11
**23**	Proximal portion of the second PP	0.26	0 (0–10)	0.12
**24**	Distal portion of the fifth PP	0.21	0 (0–10)	0.06
**25**	Distal portion of the second PP	0.14	0 (0–10)	0.06
**26**	Proximal portion of the fifth PP	0.08	0 (0–3)	0.06
**27**	Proximal portion of the third PP	0.08	0 (0–2)	0.07
**28**	Fourth MT head	0.07	0 (0–2)	0.06
**29**	Distal portion of the fourth PP	0.02	0 (0–1)	0.02
**30**	Distal portion of the third PP	0.00	0 (0–0)	0.00
**31**	Proximal portion of the fourth PP	0.00	0 (0–0)	0.00

B: RAMRIS erosion scores and prevalence of erosion of each region and joint			
**Regions and joints**	**Mean score**	**Median score**	**Prevalence**
**Forefoot**	4.86	2 (0–54)	0.79
First MTP joint	2.18	2 (0–17)	0.74
Second MTP joint	0.69	0 (0–15)	0.21
Third MTP joint	0.36	0 (0–12)	0.13
Fourth MTP joint	0.07	0 (0–2)	0.06
Fifth MTP joint	0.52	0 (0–13)	0.23
**Midfoot**	6.31	6 (0–47)	0.79
Medial cuneiform- first MT base	1.20	1 (0–10)	0.68
Middle cuneiform- second MT base	1.52	2 (0–11)	0.64
Lateral cuneiform- third MT base	1.48	1 (0–12)	0.66
Cuboid-fourth MT base	1.58	1 (0–15)	0.62
Cuboid-fifth MT base	1.20	1 (0–15)	0.61
Navicular- medial cuneiform	1.48	2 (0–10)	0.72
Navicular- middle cuneiform	1.51	2 (0–13)	0.69
**Hindfoot**	3.80	3 (0–39)	0.78
Distal tibia- proximal talus	0.95	1 (0–6)	0.54
Distal talus- proximal calcaneus	1.13	1 (0–18)	0.54
Anterior talus-navicular	1.25	1 (0–16)	0.64
Anterior process of calcaneus- cuboid	1.14	1 (0–8)	0.60

Note: grayscale lines depending on the region of the foot: light gray for the forefoot, intermediate gray for the midfoot and dark gray for the hindfoot.

Abbreviations: MT: metatarsal; MTP: metatarsophalangeal joint; PP: proximal phalanx

**Fig 3:**
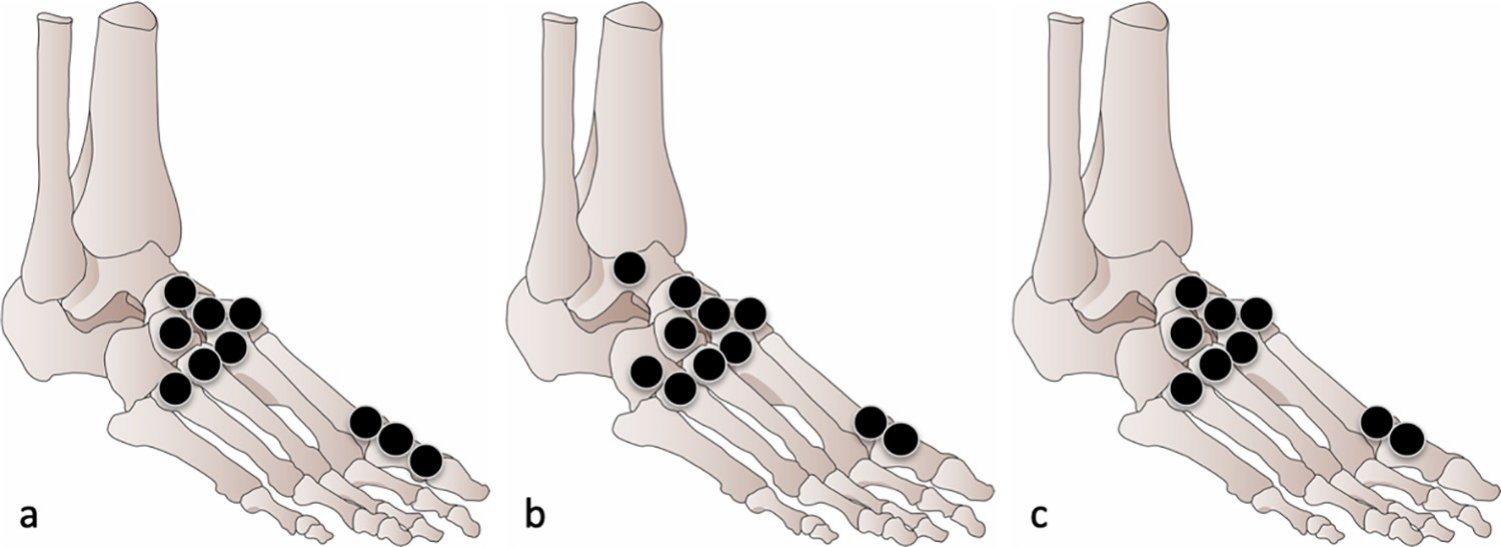
The most severely affected bone sites (Fig 3A) according to the RAMRIS-derived score and the most frequently affected sites (Fig 3B) in our study population.

Fig 3 represents the 10 most severely affected sites (Fig 3A) and the 10 most frequently affected sites (Fig 3B).

Concerning the severity of bone erosions, assessed by the mean erosion score for each site, the first MT head had the highest score (1.24), then sites belonging to the first ray (0.73 to 0.94), and then sites belonging to the midfoot (0.51 to 0.91). Except for bones of the first ray, sites of the forefoot had the lowest erosion score (0.00 to 0.44). The first metatarsophalangeal (MTP) joint had the highest MTP joint score (2.18) and the fourth MTP joint had the lowest MTP joint score (0.07). The midfoot had the highest mean erosion score (6.31), higher than the forefoot and the hindfoot (4.86 and 3.80, respectively).

Concerning the prevalence of bone erosions for each site, the first MT heads were the most frequently affected (71.0% of feet), followed by sites belonging mostly to the midfoot. Except for the first ray, sites of the forefoot had the lowest prevalence of bone erosions (0.00 to 0.21). The first MTP joint was the most often affected joint (73.6%), with 90% of patients (54/60) having at least one first MTP involved, whereas the fourth MTP joint was the least frequently affected joint (5.8%).

Distribution of erosions in the right and left feet did not significantly differ on ANOVA (p = 0.11) and Cochran-Mantel-Haenszel test (p = 0.13).

*RAMRIS-derived and Dalbeth-simplified scores*. Mean RAMRIS-derived and Dalbeth-simplified scores were 29.7 ± 35 (maximum score 236) and 10.7 ± 11 (maximum score 66), respectively (Table 3). Intra- and inter-observer agreement for the RAMRIS-derived score were excellent: ICC 0.93 (95% CI: 0.83–0.97) and 0.96 (95% CI: 0.90–0.98). ICC values were similar for the Dalbeth-simplified score: ICC 0.94 (95% CI: 0.87–0.98) and 0.90 (95% CI: 0.76–0.96).

**Table 3:** Erosion scores obtained with the RAMRIS-derived and Dalbeth-simplified scores in the study population.

Scores	Mean ± SD	Median (Q1–Q3)	Range
RAMRIS	29.7 ± 35	24.0 (8.0–38.0)	0.0–236.0
Dalbeth-simplified	10.7 ± 11	9.0 (3.0–14.0)	0.0–66.0

RAMRIS-derived score assesses 31 sites/foot. Maximum possible RAMRIS score: 620

Dalbeth-simplified score assesses 7 sites/foot. Maximum possible Dalbeth score:140

#### Correlation among clinical features, urate volume, and erosion scores

Age, serum urate, and ULT were not significantly related to erosion scores. We found no significant differences by sex for any erosion score. The RAMRIS-derived and Dalbeth-simplified scores were both significantly correlated with urate volume (r = 0.63 and 0.58, p < 0.001) and were correlated with one another (r = 0.96, p < 0.001) (Table 4).

**Table 4:** Correlations (r) between erosion RAMRIS-derived and Dalbeth-simplified scores and DECT urate volume.

	Erosion scores
	RAMRIS	Dalbeth
**Urate volume**	0.63 (0.45–0.77)*	0.58 (0.38–0.73)*
**RAMRIS**		0.96 (0.94–0.98)*

Data are correlation (r) (95% confidence intervals). *: p < 0.001.

## Discussion

We studied 61 patients with gout, with 3,751 sites scored for erosions. We highlighted several points. Except for the bones of the first ray, frequently and severely affected, the forefoot was infrequently involved, with minor erosions. The midfoot was preferentially affected over the forefoot and hindfoot in gout. Tophaceous gout was not an asymmetrical disease of the feet. Bone erosion scores were significantly correlated with the urate volume automatically determined by DECT. There was an almost perfect correlation between the two erosion scores.

The first right and left MT heads were the two most frequently affected bones (prevalence 71.7% for the right and 69.5% for the left). The base of the first phalanges was less frequently involved than the first MT head (prevalence 62.7% for the right, 49.2% for the left). Overall, 90% of our patients had at least one first MTP joint involved. This result agrees with clinical studies that have long reported that first MTP was the most frequently affected joint in gout [28–32] and with a recent meta-analysis of 11 studies providing an estimated average prevalence of 73% for first-MTP arthritis across studies [33]. Our erosion prevalence was higher (90%), probably because we took into account erosions at imaging, symptomatic or not. The first MTP was also severely affected. Indeed, this joint had the highest mean erosion score (2.18), with the first MT head and the base of the first phalanx having the two highest mean bone erosion scores (1.24 and 0.94, respectively). Patients with first MTP tophaceous gout have high levels of pain and altered gait patterns with slow walking velocity [33, 34].

Except for bones of the first ray, the forefoot was infrequently involved, and erosions were not severe. The fourth MTP joint was the least frequently affected joint and had the lowest RAMRIS-derived erosion score, in line with a previous radiographic study that mentioned sparing of this joint [32]. We also noted that the erosion score decreased from the first to the fourth MTP joints, then increased again at the fifth MTP joint. Mechanical factors might explain the predilection of gout for the first and fifth MTP joints. First, the architecture of the foot with the inner and outer arches of the foot put pressure points on these two MTPs, with the forward transfer of body weight during propulsion largely implicating the first MTP [33], with frequent osteoarthritic cartilage lesions [35]. Second, physical shocks, believed to favour MSU crystal formation [36], are more frequent at the inner and outer border of the foot. Thermal factors could also explain the predilection of urate deposits at the first and fifth MTP joints with lower temperatures decreasing the solubility of urate [37, 38].

In our CT study, the midfoot was frequently affected by tophaceous gout, more than the forefoot and hindfoot regions. The CT technique, with reformatted images in the three planes, is a powerful tool to assess erosions of the midfoot. Doyle et al. [39] and Dalbeth et al. [17] had a similar finding with conventional CT images. *A contrario* standard radiography is inherently less sensitive because of bone superimposition. The midfoot belongs to the transverse arch and supports a mechanical load that could participate in the genesis of deposits and structural damage. Moreover, patients with chronic gout have higher mid-foot pressure-time integrals than do controls, probably because of the redistribution of the plantar support points related to their altered gait pattern with an attempt to off-load the first MTP joint [35].

In our study, both the prevalence and erosion score did not significantly differ between right and left sides, in contrast to the classical view that gout is an asymmetrical arthritis [40–43]. Our findings confirm those of Doyle et al. [39] and Yokose et al. [14] who recently suggested that erosive disease from gout was, in fact, a symmetric process. However, because of the cross-sectional design of our study, we cannot rule out that erosions could be asymmetrical in early gout and evolve with time to a symmetrical disease.

Bone erosion scores, RAMRIS-derived and Dalbeth-simplified scores, are mostly used in studies and not in current clinical practice because of the long time to obtain and complexity for non-trained radiologists. Yet the Dalbeth-simplified score is faster and easier to obtain. Our results highlighted several points. First there was an excellent intra- and inter-observer agreement for the RAMRIS-derived and Dalbeth-simplified scores. Second the results showed that the seven sites analyzed in the Dalbeth-simplified score (i.e., the first MT head; the second, third and fourth MT bases; cuboid, middle cuneiform, and distal tibia [not studied here]) are included in our 10 sites with the highest prevalence of erosions. Third there was an almost perfect correlation between the two erosion scores. These points provide reassurance that the simplified scoring system is performing well. In our study, the RAMRIS-derived and Dalbeth-simplified scores were both significantly correlated with urate volume (r = 0.58–0.63) with r values similar to those obtained in a previous study [25]. However despite these good correlations, DECT urate volume, which is automatically calculated and therefore easy to obtain and reproducible, can not be a marker of bone destruction in patients with ULT. Indeed there is a lag between MSU crystal dissolution and stabilization or improvement of structural damage [25].

Our study has some limitations. We analyzed the feet and ankles of 61 patients, which may seem a relatively small population. However, our sample consisted exclusively of patients with gout who had a DECT-positive detection of MSU crystal deposits at the feet and ankle, and we scored 3,751 bones for erosion, which, to our knowledge, is unparalleled in the literature. We did not evaluate the tophus locations, especially by differentiating tophi contacting bone or distant from bone, and correlation with erosion scores. DECT studies [44–46] have revealed frequent tendinous locations in patients with tophaceous gout, especially at the feet, that probably also contribute to impaired function. Finally even if the reader was blinded to the DECT urate volume, since the erosions were evaluated on the CT images, there is a possibility that the reader has been affected when evaluating the erosions.

To summarize, DECT allows for reconstructing high-resolution CT images of bone that can be used to analyze erosions, and automatically determine urate volume. We precisely report bone erosion distribution in the feet of patients with gout and describe the correlation between urate volume automatically determined by DECT and two erosion scores. The RAMRIS-derived and Dalbeth-simplified scores were both significantly correlated with urate volume. Finally there was an excellent correlation between the two scores, which suggests that the simplified score can be used with confidence.

## Supporting information

S1 Table Parameters used for image acquisition and reconstruction. (DOCX)

## References

[1] Bardin T, Richette P. Impact of comorbidities on gout and hyperuricaemia: an update on prevalence and treatment options. BMC Med 2017;15:123. doi: 10.1186/s12916-017-0890-9 28669352 PMC5494879

[2] Bongartz T, N Glazebrook K, J Kavros S, et al. Dual-energy CT for the diagnosis of gout: An accuracy and diagnostic yield study. Ann Rheum Dis 2014;74:1072–1077. doi: 10.1136/annrheumdis-2013-205095 24671771 PMC4431329

[3] Choi HK, Al-Arfaj AM, Eftekhari A, et al. Dual energy computed tomography in tophaceous gout. Ann Rheum Dis 2009;68:1609–1612. doi: 10.1136/ard.2008.099713 19066180

[4] Choi HK, Burns LC, Shojania K, et al. Dual energy CT in gout: a prospective validation study. Ann Rheum Dis 2012;71:1466–1471. doi: 10.1136/annrheumdis-2011-200976 22387729

[5] Glazebrook KN, Guimarães LS, Murthy NS, et al. Identification of Intraarticular and Periarticular Uric Acid Crystals with Dual-Energy CT: Initial Evaluation. Radiology 2011;261:516–524. doi: 10.1148/radiol.11102485 21926378

[6] Hu H-J, Liao M-Y, Xu L-Y. Clinical utility of dual-energy CT for gout diagnosis. Clin Imaging 2015;39:880–885. doi: 10.1016/j.clinimag.2014.12.015 25725947

[7] Ahmad Z, Gupta A, Sharma R, et al. Dual Energy Computed Tomography: A Novel Technique for Diagnosis of Gout. Int J Rheum Dis 2016;19:887–96. doi: 10.1111/1756-185X.12874 27125882

[8] Breuer G, Bogot N, Nesher G. Dual-energy Computed Tomography as a Diagnostic Tool for Gout During Intercritical Periods. Int J Rheum Dis 2016;19:1337–1341. doi: 10.1111/1756-185X.12938 27458073

[9] Huppertz A, Hermann K-GA, Diekhoff T, et al. Systemic staging for urate crystal deposits with dual-energy CT and ultrasound in patients with suspected gout. Rheumatol Int 2014;34:763–771. doi: 10.1007/s00296-014-2979-1 24619560

[10] Ogdie A, Taylor W, Weatherall M, et al. Imaging Modalities for the Classification of Gout: Systematic Literature Review and Meta-Analysis. Ann Rheum Dis 2015;74:1868–74. doi: 10.1136/annrheumdis-2014-205431 24915980 PMC4869978

[11] Omoumi P, Verdun FR, Guggenberger R, et al. Dual-Energy CT: Basic Principles, Technical Approaches, and Applications in Musculoskeletal Imaging (Part 2). Semin Musculoskelet Radiol 2015;19:438–445. doi: 10.1055/s-0035-1569252 26696082

[12] Shi D, Xu J-X, Wu H-X, et al. Methods of assessment of tophus and bone erosions in gout using dual-energy CT: reproducibility analysis. Clin Rheumatol 2015;34:755–765. doi: 10.1007/s10067-014-2725-9 24935412

[13] Shi D, Chen J-Y, Wu H-X, et al. Relationship between urate within tophus and bone erosion according to the anatomic location of urate deposition in gout: A quantitative analysis using dual-energy CT volume measurements. Medicine 2019;98:e18431. doi: 10.1097/MD.0000000000018431 31861011 PMC6940130

[14] Yokose C, Dalbeth N, Wei J, et al. Radiologic evidence of symmetric and polyarticular monosodium urate crystal deposition in gout—A cluster pattern analysis of dual-energy CT. Semin Arthritis Rheum 2020;50:54–58. doi: 10.1016/j.semarthrit.2019.07.002 31371194 PMC6954342

[15] McQueen FM, Doyle A, Reeves Q, et al. Bone erosions in patients with chronic gouty arthropathy are associated with tophi but not bone oedema or synovitis: new insights from a 3 T MRI study. Rheumatol 2014;53:95–103. doi: 10.1093/rheumatology/ket329 24080252

[16] Døhn UM, Ejbjerg BJ, Hasselquist M, et al. Rheumatoid arthritis bone erosion volumes on CT and MRI: reliability and correlations with erosion scores on CT, MRI and radiography. Ann Rheum Dis 2007;66:1388–1392. doi: 10.1136/ard.2007.072520 17606464 PMC1994287

[17] Dalbeth N, Doyle A, Boyer L, et al. Development of a computed tomography method of scoring bone erosion in patients with gout: validation and clinical implications. Rheumatology 2011;50:410–416. doi: 10.1093/rheumatology/keq335 21059673

[18] Østergaard M, Peterfy C, Conaghan P, et al. OMERACT Rheumatoid Arthritis Magnetic Resonance Imaging Studies. Core set of MRI acquisitions, joint pathology definitions, and the OMERACT RA-MRI scoring system. J Rheumatol 2003;30:1385–1386 12784422

[19] Østergaard M, Peterfy CG, Bird P, et al. The OMERACT Rheumatoid Arthritis Magnetic Resonance Imaging (MRI) Scoring System: Updated Recommendations by the OMERACT MRI in Arthritis Working Group. J Rheumatol 2017;44:1706–1712. doi: 10.3899/jrheum.161433 28811353

[20] Dalbeth N, Clark B, Gregory K, et al. Mechanisms of bone erosion in gout: a quantitative analysis using plain radiography and computed tomography. Ann Rheum Dis 2009;68:1290–1295. doi: 10.1136/ard.2008.094201 18708415

[21] Towiwat P, Doyle AJ, Gamble GD, et al. Urate crystal deposition and bone erosion in gout: ‘inside-out’ or ‘outside-in’? A dual-energy computed tomography study. Arthritis Res Ther 2016;18:208. doi: 10.1186/s13075-016-1105-z 27629724 PMC5024428

[22] Sapsford M, Gamble GD, Aati O, et al. Relationship of bone erosion with the urate and soft tissue components of the tophus in gout: a dual energy computed tomography study. Rheumatol 2017;56:129–133. doi: 10.1093/rheumatology/kew383 27803304

[23] Towiwat P, Chhana A, Dalbeth N. The anatomical pathology of gout: a systematic literature review. BMC Musculoskelet Disord 2019;20:140. doi: 10.1186/s12891-019-2519-y 30935368 PMC6444644

[24] Dalbeth N, Smith T, Nicolson B, et al. Enhanced osteoclastogenesis in patients with tophaceous gout: urate crystals promote osteoclast development through interactions with stromal cells. Arthritis Rheum 2008;58:1854–1865. doi: 10.1002/art.23488 18512794

[25] Dalbeth N, Billington K, Doyle A, et al. Effects of Allopurinol Dose Escalation on Bone Erosion and Urate Volume in Gout: A Dual-Energy Computed Tomography Imaging Study Within a Randomized, Controlled Trial. Arthritis Rheumatol 2019;71:1739–1746. doi: 10.1002/art.40929 31081595

[26] Neogi T, Jansen TLTA, Dalbeth N, et al. Gout Classification Criteria: an American College of Rheumatology/European League Against Rheumatism collaborative initiative. Arthritis Rheumatol 2015;67:2557–2568. doi: 10.1002/art.39254 26352873 PMC4566153

[27] Landis JR, Koch GG. The measurement of observer agreement for categorical data. Biometrics 1977;33:159–174 843571

[28] Grahame R, Scott JT. Clinical survey of 354 patients with gout. Ann Rheum Dis 1970;29:461–468. doi: 10.1136/ard.29.5.461 5476673 PMC1010557

[29] Janssens HJEM, Janssen M, van de Lisdonk EH, et al. Use of oral prednisolone or naproxen for the treatment of gout arthritis: a double-blind, randomised equivalence trial. Lancet 2008;371:1854–1860. doi: 10.1016/S0140-6736(08)60799-0 18514729

[30] Roddy E, Zhang W, Doherty M. Are joints affected by gout also affected by osteoarthritis? Ann Rheum Dis 2007;66:1374–1377. doi: 10.1136/ard.2006.063768 17284542 PMC1994292

[31] Garcia CO, Kutzbach AG, Espinoza LR. Characteristics of gouty arthritis in the Guatemalan population. Clin Rheumatol 1977;16:45–50. 10.1007/BF02238762 9132325

[32] Zeng Q, Wang Q, Chen R, et al. Primary Gout in Shantou: A Clinical and Epidemiological Study. Chin Med J 2003;116:66–9 12667391

[33] Stewart S, Dalbeth N, Vandal AC, Rome K. The first metatarsophalangeal joint in gout: a systematic review and meta-analysis. BMC Musculoskelet Disord 2016;17:69. doi: 10.1186/s12891-016-0919-9 26864742 PMC4750194

[34] Rome K, Survepalli D, Sanders A, et al. Functional and biomechanical characteristics of foot disease in chronic gout: A case-control study. Clin Biomech 2011;26:90–94. doi: 10.1016/j.clinbiomech.2010.09.006 20950904

[35] Neogi T, Krasnokutsky S, Pillinger MH. Urate and Osteoarthritis: Evidence For a Reciprocal Relationship. Jt Bone Spine 2019;86:576–582. doi: 10.1016/j.jbspin.2018.11.002 30471419 PMC6531371

[36] Wilcox WR, Khalaf AA. Nucleation of monosodium urate crystals. Ann Rheum Dis 1975;34:332–339 doi: 10.1136/ard.34.4.332 242279 PMC1006423

[37] Kippen I, Klinenberg JR, Weinberger A, Wilcox WR. Factors affecting urate solubility in vitro. Ann Rheum Dis 1974;33:313–317. doi: 10.1136/ard.33.4.313 4413418 PMC1006264

[38] Loeb JN. The influence of temperature on the solubility of monosodium urate. Arthritis Rheum 1972;15:189–192. doi: 10.1002/art.1780150209 5027604

[39] Doyle AJ, Dalbeth N, McQueen F, et al. Gout on CT of the feet: A symmetric arthropathy: Gout symmetry. J Med Imaging Radiat Oncol 2016;60:54–58. doi: 10.1111/1754-9485.12419 26631920

[40] Monu JUV, Pope TL. Gout: a clinical and radiologic review. Radiol Clin North Am 2004;42:169–184. doi: 10.1016/S0033-8389(03)00158-1 15049530

[41] Watt I, Middlemiss H. The radiology of gout. Review article. Clin Radiol 1975;26:27–36. doi: 10.1016/s0009-9260(75)80004-3 804372

[42] Gentili A. Advanced imaging of gout. Semin Musculoskelet Radiol 2003;7:165–174. doi: 10.1055/s-2003-43227 14593558

[43] Dhanda S, Jagmohan P, Quek ST, Tian QS. A re-look at an old disease: a multimodality review on gout. Clin Radiol 2011;66:984–992. doi: 10.1016/j.crad.2011.04.011 21658689

[44] Bayat S, Aati O, Rech J, et al. Development of a Dual-Energy Computed Tomography Scoring System for Measurement of Urate Deposition in Gout. Arthritis Care Res 2016;68:769–75. doi: 10.1002/acr.22754 26474153

[45] Mallinson PI, Reagan AC, Coupal T, et al. The distribution of urate deposition within the extremities in gout: a review of 148 dual-energy CT cases. Skeletal Radiol 2014;43:277–281. doi: 10.1007/s00256-013-1771-8 24337414

[46] Dalbeth N, Kalluru R, Aati O, et al. Tendon Involvement in the Feet of Patients With Gout: A Dual-Energy CT Study. Ann Rheum Dis 2013;72:1545–1548. doi: 10.1136/annrheumdis-2012-202786 23334212

