# Supplementary material for: Distribution of bony erosions in feet and performance of two bone erosion scores: A dual-energy computed tomography study of 61 patients with gout
Source: PLoS One. 2021 Nov 2;16(11):e0259194. doi: 10.1371/journal.pone.0259194 (PMC8562819; doi:10.1371/journal.pone.0259194)
Supplement: S1 Table — (DOCX) [file pone.0259194.s001.docx]

**S1 Table. Parameters used for image acquisition and reconstruction**

| **Parameters used for image acquisition** | |
| --- | --- |
| Mode | Helical |
| kV / mA | **80** / 600 |
|  | **135** / 150 |
| Detector configuration (mm) | 0.5 x 80 |
| FOV (mm) | 320 |
| Pitch | 0.64 |
| Rotation time (sec) | 0.5 |
| Slice thickness (mm) | 0.5 |
|  |  |
|  |  |
| **Parameters used for image reconstruction** | |
| FOV (mm) | 280 |
| Kernels used for reconstruction | FC 17 (soft) +FC 30 (hard) |
| Slice thickness (mm) | 0.5 |
| Reconstruction increment (mm) | 0.3 |
| Matrix | 512x512 |
| Iterative reconstruction | AIDR 3D Strong |

Abbreviations: FOV: field of view; AIDR 3D: adaptative iterative dose reduction
